# Changes in Prehospital Stroke Care and Stroke Mimic Patterns during the COVID-19 Lockdown

**DOI:** 10.3390/ijerph18042150

**Published:** 2021-02-23

**Authors:** Kazimieras Melaika, Lukas Sveikata, Adam Wiśniewski, Altynshash Jaxybayeva, Aleksandra Ekkert, Dalius Jatužis, Rytis Masiliūnas

**Affiliations:** 1 Faculty of Medicine, Vilnius University, 03101 Vilnius, Lithuania; k.melaika@gmail.com; 2 J. Philip Kistler Stroke Research Center, Department of Neurology, Massachusetts General Hospital, Harvard Medical School, Boston, MA 02109, USA; lsveikata@mgh.harvard.edu; 3 Institute of Cardiology, Medical Academy, Lithuanian University of Health Sciences, 50161 Kaunas, Lithuania; 4 Department of Neurology, Collegium Medicum in Bydgoszcz, Nicolaus Copernicus University in Torun, 85-094 Bydgoszcz, Poland; adam.lek@wp.pl; 5 Department of Neurology, Astana Medical University, Nur-Sultan 010000, Kazakhstan; altynshash@gmail.com; 6 Center of Neurology, Vilnius University, 08661 Vilnius, Lithuania; aleksandra.ekkert@mf.vu.lt (A.E.); dalius.jatuzis@mf.vu.lt (D.J.)

**Keywords:** COVID-19, emergency medical services, stroke, misdiagnosis, stroke mimic, triage

## Abstract

The impact of COVID-19 lockdown on prehospital stroke care is largely unknown. We aimed to compare stroke care patterns before and during a state-wide lockdown. Thus, we analysed prospective data of stroke alerts referred to our stroke centre between 1 December 2019 and 16 June 2020, and compared them between two periods—15 weeks before and 13 weeks during the state-wide lockdown declared in Lithuania on 16 March 2020. Among 719 referrals for suspected stroke, there was a decrease in stroke alerts (rate ratio 0.61, 95% CI (0.52–0.71)), stroke admissions (0.63, 95% CI (0.52–0.76)), and decrease in prehospital stroke triage quality (positive predictive value 72.1% vs. 79.9%, *p* = 0.042) during the lockdown. The onset-to-door time was longer (153.0 vs. 120.5 min, *p* = 0.049) and seizures and intracranial tumours were more common among stroke mimics (16.9% vs. 6.7%, *p* = 0.012 and 9.6% vs. 3.0%, *p* = 0.037, respectively). We conclude that there was a decline in prehospital stroke triage quality during the lockdown despite low COVID-19 incidence in the country. Moreover, we observed an increase in hospital arrival delays and severe conditions presenting as stroke mimics. Our findings suggest that improved strategies are required to maintain optimal neurological care during public health emergencies.

## 1. Introduction

Emergency medical services (EMS) are the first healthcare contact for most stroke patients [1,2] and play a crucial role in identifying acute stroke [3]. Accurate recognition and timely transport of patients with suspected stroke to comprehensive stroke centres (CSCs) are closely correlated with acute stroke care success [2,4]. Any delay in administering intravenous thrombolysis (IVT) and endovascular treatment (EVT) negatively impacts patients’ functional outcomes [5].

The typical presentation of stroke consists of a sudden onset of a focal neurological deficit. However, other disorders may also have similar clinical presentations. These false positives, called stroke mimics, comprise from 15% to 37% of suspected stroke patients at the Emergency Department (ED) [6,7]. The consequences of stroke mimics may result in inappropriate usage of stroke care facilities and medical resources and increased workload on overwhelmed ED personnel [6,8]. A decreasing number of stroke admissions observed during the ongoing severe acute respiratory coronavirus 2 (SARS-CoV-2) outbreak causing the coronavirus disease 2019 (COVID-19) pandemic raised the concern of suboptimal prehospital identification and referral of acute illnesses [9,10,11,12].

The substantial strain imposed by the pandemic on the medical systems worldwide caused significant concern regarding the potential ramifications on acute stroke care [13,14]. Soon after identifying the first COVID-19 case in Lithuania, a strict national lockdown was declared on 16 March 2020 [15]. Interestingly, in Lithuania, the community spread and deaths due to COVID-19 during the first wave of the pandemic remained exemplarily low (a total of 1775 infections and 76 deaths as of 16 June 2020) [16]. However, strict restrictions on public life and reduced access to healthcare services, including primary care and preventive programs [15], as well as public fear of coronavirus, might have affected stroke care. Understanding the impact of state-wide lockdown measures on acute stroke care is crucial to improving healthcare systems’ coordination during public health emergencies. There are currently few studies evaluating stroke care in low COVID-19 incidence settings [17,18] and no studies addressing the impact on prehospital stroke triage.

To assess the impact of COVID-19 lockdown on prehospital stroke care and referral patterns, we analysed data from our stroke care network. The aim of our study was (1) to compare the accuracy of the prehospital identification of acute stroke among different healthcare providers, (2) to assess stroke incidence and acute care timeliness metrics, and (3) to evaluate the distribution of stroke mimics before and during the state-wide COVID-19 lockdown. We hypothesised that lockdown measures would be associated with decreased stroke triage quality, increased delays in stroke care, and changes in the pattern of conditions mimicking stroke.

## 2. Methods

### 2.1. Study Design

**Figure 1:**
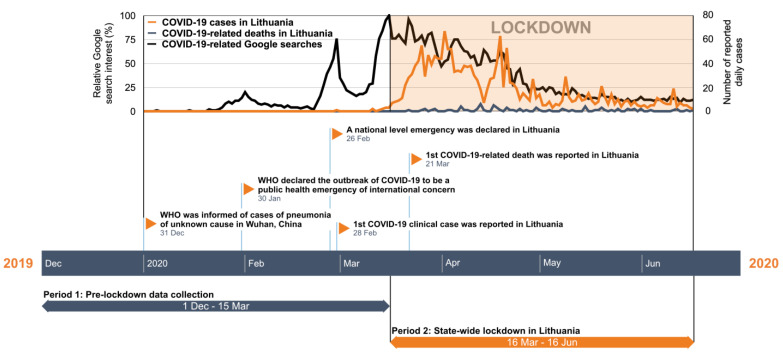
COVID-19 public concern and study timeline. Timeline of data collection periods overlapped with normalised data from COVID-19 related Google searches in Lithuania (100—high interest; 0—no or insufficient interest data) and COVID-19 daily incidence as reported by the Lithuanian National Public Health Center. COVID-19, coronavirus disease 2019; WHO, World Health Organization.

A single-centre prospective observational study was carried out on prehospital stroke triage quality between 1 December 2019 and 16 June 2020, at Vilnius University Hospital (VUH)—one of the two comprehensive stroke centres in Eastern Lithuania with a catchment population of 945,000 inhabitants. The collected data were compared among two equal periods—15 weeks before and 13 weeks during a state-wide lockdown declared on 16 March 2020 (Figure 1) [15].

The study was approved by a regional bioethics committee and complies with STROBE guidelines for observational research [19].

### 2.2. Study Population

**Figure 2:**
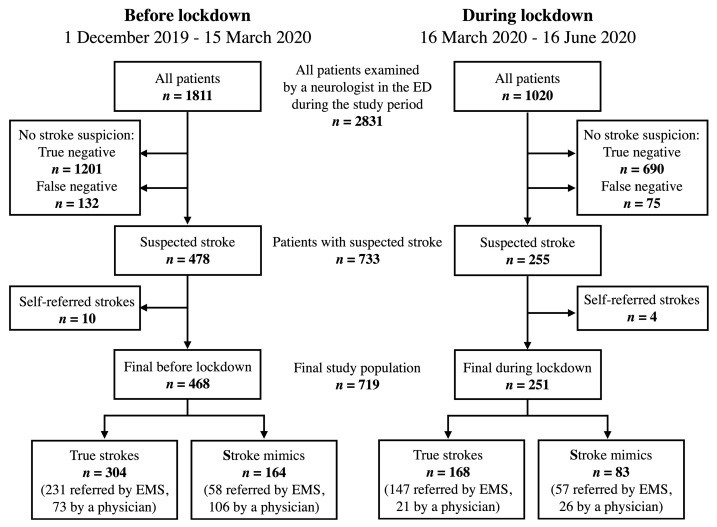
Flowchart of study population: patients examined by a neurologist in the Emergency Department (ED) of Vilnius University Hospital between 1 December 2019 and 16 June 2020. ED, emergency department; EMS, emergency medical services.

All patients referred to the VUH Emergency Department either by EMS or an outpatient physician with a prehospital diagnosis of suspected stroke or transient ischaemic attack (TIA) were included. A specialist neurologist in the ED ascertained stroke cases. Study flowchart of patients included in the study can be seen in Figure 2.

Throughout Lithuania, emergency medical care is provided free of charge 24/7 to everyone, regardless of health insurance status. EMS staff includes a certified medical specialist—a nurse or a paramedic—who decides whether a patient needs to be taken to a CSC. Face Arm Speech Test (FAST) is uniformly used for the identification of a suspected stroke/TIA [3]. Alternatively, any primary care or specialist physician can refer a patient directly to the ED, in case of suspected stroke.

### 2.3. Data Collection

Demographic and clinical characteristics such as age, sex, stroke type, National Institutes of Health Stroke Scale (NIHSS) scores at admission and discharge, number and type of stroke mimics, reperfusion therapy, and acute stroke care timeliness metrics were collected for all stroke alerts. The NIHSS score was documented only for patients who were considered for reperfusion therapy. We compared the variables and positive predictive value (PPV) for the correct stroke identification among the EMS and referring physicians before and during the state-wide lockdown.

Public concern for COVID-19 was evaluated based on the relative Lithuanian Google search interest of the five most common COVID-19-related terms—“corona”, “korona“, “koronavirusas“, “coronavirus“, and “COVID“, publicly available through Google Trends [20]. Google Trends has emerged as a useful tool to measure public concern of SARS-CoV-2 infection in the population [21]. Search query data was collected from Google Trends and was normalised to reflect the interest, expressed by 100 as high interest and lack of interest or insufficient data as 0. Lithuanian National Public Health Center data on daily COVID-19 cases and patient mortality were used to illustrate the magnitude of the COVID-19 pandemic in Lithuania [16].

### 2.4. Statistical Analysis

A Student’s *t*-test and Mann–Whitney U test was used to compare quantitative variables, as appropriate. For categorical variables, the Chi-square test and Fisher’s exact test were used, as appropriate. Individuals were further categorised into groups by referral type (EMS and referring physician) and periods (before and during the lockdown). At first, we evaluated the stroke presentation rates during different periods and different providers. We then used Poisson regression models with the daily counts of stroke alerts and stroke admissions as dependent variables and the study period as an independent variable. Second, we evaluated the stroke care metrics and the distribution of stroke mimics between different healthcare providers and periods. To assess the stroke triage quality, we calculated the positive predictive value (PPV) of acute stroke identification for each group with 95% confidence intervals (CI) and compared them before and during the lockdown. *p* < 0.05 (two-sided) was considered to be statistically significant. IBM SPSS Statistics 23.0 software (Armonk, NY, USA: IBM Corp) was used for statistical analyses.

## 3. Results

### 3.1. Demographic and Clinical Characteristics

In total, 719 patients with suspected stroke were included in our analysis: 493 referred by EMS and 226 by outpatient physicians (Figure 2). Patients did not differ significantly in sex and age, regardless of being referred by EMS or a physician (Table 1). None of the patients were diagnosed with COVID-19.

We observed significantly more daily stroke alerts and confirmed strokes in the EMS group compared to the outpatient physicians’ group (2 [1–4] and 2 [1–2] vs. 1 [0–2] and 0 [0–1], respectively, *p* < 0.001). There were significantly fewer stroke mimics in the EMS group (23.3% vs. 58.4%, *p* < 0.001).

The median onset-to-door (OTD) time was twice as short in patients referred by EMS (116 min vs. 245, *p* < 0.001), however, the door-to-needle (DTN) time and the door-to-groin (DTG) time did not differ significantly between the groups. The baseline and discharge NIHSS scores also did not differ significantly, but there was a tendency for lower baseline NIHSS in the physicians’ group (8 (4–15) vs. 6.5 (3–12), *p* = 0.175).

There were more patients with ischaemic stroke eligible for reperfusion therapy in the group referred by EMS (31.5% vs. 9.2%, *p* < 0.001). This significance was driven by a higher proportion of patients eligible for IVT (15.3% vs. 1.3%, *p* < 0.001). However, the eligibility for EVT (10.9% vs. 6.6%, *p* = 0.395) or combined treatment (5.3% vs. 1.3%, *p* = 0.217) did not differ significantly between the groups.

### 3.2. Lockdown Data

There were no significant differences in age, sex, the proportion of stroke mimics, percentage of patients eligible for reperfusion therapy, median baseline and discharge NIHSS scores, median DTN or DTG time between patients admitted in the period before and during the lockdown. However, we found fewer patients with TIA (2.4% vs. 5.8%, *p* = 0.039), and longer median OTD time in patients admitted during the lockdown (153.0 vs. 120.5 min, *p* = 0.049). We also observed a decrease in the median daily volume of stroke alerts (3 [1–4] vs. 4 [3–6], *p* < 0.001) and confirmed strokes (2 [1–2] vs. 3 [2–4], *p* < 0.001) during the lockdown. Strikingly, during the lockdown, there was a threefold decrease in stroke referrals by outpatient physicians. However, the prevalence of confirmed strokes in the ED during the period before and during the state-wide lockdown remained stable (24.6% vs. 24.2%, *p* = 0.807) (Figure 2).

Poisson regression revealed that the lockdown period was associated with a reduction in daily stroke alerts with a rate ratio of 0.61 (95% CI 0.52–0.71, *p* < 0.001) and stroke admissions by 0.63 (95% CI 0.52–0.76, *p* < 0.001).

### 3.3. Prehospital Stroke Triage Quality

The positive predictive value (PPV) for identifying patients with acute stroke or TIA by EMS was significantly lower during the lockdown than the period before (72.1% vs. 79.9%, *p* = 0.042). However, the PPV did not change in the outpatient physicians’ group (44.7% vs. 40.8%, *p* = 0.629). The overall PPV for acute strokes was significantly higher in the EMS group than the outpatient physicians’ group (76.7% vs. 41.6%, *p* < 0.001) (Table 2).

### 3.4. Public Concern for COVID-19

As reflected by the relative Lithuanian Google search interest of the COVID-19-related terms, the coronavirus concern increased just before the national-level emergency was declared in Lithuania on 26 February 2020 and peaked at the start of the state-wide lockdown on 16 March 2020 (Figure 1). At that point, only 12 patients were diagnosed with COVID-19 overall in the country, and none had died from the disease [16]. In general, throughout the study period, the incidence and mortality of COVID-19 in Lithuania remained one of the lowest in Europe: 63.8 vs. 264.7/100,000 cumulative cases and 2.7 vs. 29.7/100,000 cumulative deaths by 16 June 2020 in Lithuania vs. the European Union, respectively [22].

### 3.5. Stroke Mimics

Seizures and infection/sepsis were significantly more common stroke mimics among those referred by EMS compared to the physicians (16.5% vs. 4.5% and 13.9% vs. 4.5%, *p* = 0.002 and *p* = 0.010, respectively), whereas peripheral vestibulopathies were significantly less common (0.9% vs. 18.0%, *p* < 0.001).

When comparing the two periods, isolated neurological symptoms associated with normal neurological work-up results were less frequent stroke mimics during the lockdown (2.4% vs. 11.6%, *p* = 0.015), whereas seizures and intracranial tumours were significantly more frequent (16.9% vs. 6.7% and 9.6% vs. 3.0%, *p* = 0.012 and *p* = 0.037, respectively) (Table 3).

## 4. Discussion

In this prospective study in an academic stroke centre with a large urban catchment population, we found a significant decrease in prehospital stroke triage quality and longer delays from symptom onset to hospital arrival during a state-wide COVID-19 lockdown. We also found a decreased number of stroke alerts and stroke admissions during the lockdown. In addition, serious neurological conditions, such as seizures and intracranial tumours, were encountered more often as stroke mimics. Our findings provide novel knowledge on the impact of the state-wide lockdown on prehospital stroke care in low COVID-19 incidence settings and help guide care delivery strategies during public health emergencies.

Although in our study, the overall 76.7% stroke identification accuracy by EMS was on the higher end compared to 64–78% reported in previous studies [3,23,24], we found that the PPV for acute stroke decreased during the lockdown in the EMS group. Although Lithuania has not experienced a significant surge of COVID-19 during the lockdown, we observed a significant increase in COVID-19 concern in the public, as indicated by Google Trends data on COVID-19 related searches. We speculate that the latter might have influenced prehospital stroke care and the decreased stroke triage quality could have in part resulted from the fear of COVID-19 exposure and EMS staff’s hesitation to perform a thorough neurological evaluation rather than relocation of healthcare resources. Besides, the PPV could have decreased due to the barriers from personal protective equipment, such as performing language evaluation with a surgical mask or a respirator [25]. On the other hand, all routine medical activities and surgeries during the state-wide lockdown were halted, resulting in a deterioration of preexisting neurological conditions [15]. Suboptimal routine care was reflected by a sharp decline in outpatient physician referrals of suspected stroke and an overall increase in the proportion of seizures and intracranial tumours mimicking stroke. Despite a significant decrease in the absolute numbers of daily stroke alerts and confirmed strokes during the lockdown, the prevalence of confirmed strokes in the ED did not differ between the periods, suggesting an effective reduction in PPV.

Decreased stroke triage quality has clinical implications during a public health crisis. More patients without the need for urgent time-sensitive care are delivered to the ED as stroke alerts, thus, increasing the workload on the ED personnel. Secondly, patients and personnel may be increasingly exposed to the risk of SARS-CoV-2 infection. Finally, continuous medical education is crucial even during a pandemic. Remote stroke care training and electronic learning modules can help ensure maintenance and adaptability of prehospital stroke care during public health emergencies [26].

In addition, the PPV for confirmed stroke was significantly higher among patients referred by the EMS rather than those referred by physicians. In contrast, a meta-analysis composed of 23 studies and 8839 patients found that the proportion of confirmed strokes was higher among the primary care (72%, 95% CI 58–86) than the ambulance referrals (55%, 95% CI 1–108) [27]. Nonetheless, the wide confidence intervals suggest that the proportion of correctly identified stroke cases vary greatly between different healthcare systems. For example, two studies in neighbouring Poland, a country with a similar primary care system, showed a resembling PPV and stroke mimic pattern [7,28]. One reason behind the difference in PPV between EMS and outpatient physicians could be a striking number of peripheral vestibulopathies being referred to the ED as stroke mimics by the outpatient physicians (18.0% vs. 0.9% of all stroke mimics, *p* < 0.001). In line with our findings, a previous study confirmed that peripheral vertigo is largely misdiagnosed in the primary care setting [29]. On the other hand, the lower diagnostic accuracy of stroke could pinpoint the lack of neurology training in the primary care and the ED serving as a pathway for a patient to receive an urgent neurology consultation. Finally, FAST screening is mandatory for EMS personnel to evaluate stroke alerts in Lithuania [3,30]. In contrast, outpatient physicians refer patients based on clinical suspicion of stroke, which carries a greater risk of false positives.

Second, we found a significantly increased delay from stroke symptom onset to hospital arrival during the lockdown. In contrast, ED stroke care delivery timeliness, such as DTN and DTG times, did not differ before and during the lockdown. Studies in Spain, one of the most heavily stricken countries by COVID-19, found a delay in prehospital and DTN times [10,31]. These findings partially differ from the analyses of major stroke care networks in the United States. Two studies found decreased stroke/TIA admission rates but no change in prehospital and ED stroke care delivery timeliness [12,32]. Similar to our study, a survey of stroke centres in China showed a 45% reduction in TIA and 32% in stroke admissions in mild epidemic regions, as well as overall increased delays to acute stroke care [33]. Although the reasons for increased onset-to-door delays are unclear, fear of virus exposure in the ambulance or at the hospital and absence of bystanders to initiate the stroke alarm during social distancing mandate might have delayed initiation of the EMS call [34]. Also, the need to perform additional respiratory screening and use of personal protective equipment could increase prehospital delays. Another possibility could be the saturation of ambulance services or stroke alerts referred to other hospitals before being transferred to a stroke centre [10].

In line with other reports from China, Europe, Taiwan, and the United States, we also found a significant decrease in stroke alerts and stroke admissions [9,11,12,17,18,31,32]. Our findings provide additional knowledge on the effects of lockdown measures on stroke care patterns in a low-incidence low-mortality COVID-19 outbreak setting. Similarly, another study of admission rates at three tertiary university hospitals in Greece, where COVID-19 penetration was also low, showed a remarkable decrease in stroke and acute coronary artery syndrome patients [17]. We also found a decreased number of TIAs during the lockdown. Thus, the reduced number of stroke/TIA consultations might be attributable to the reluctance to seek medical attention in the presence of mild or transient neurological symptoms. Even though our study did not find a significant change in presenting stroke severity, we might have been underpowered due to missing NIHSS in patients not considered for reperfusion.

Thirdly, we found that seizures and intracranial tumours presented more often as stroke mimics during the lockdown. A recent study from our institution showed that worse seizure control and health status in people with epilepsy were observed during the state-wide lockdown [35]. Similarly, a study from the United Kingdom reported that delays in cancer diagnosis and care are projected to result in more than 3200 avoidable cancer deaths in the following five years [36]. Altogether, these findings underscore the importance of interventions to optimise specialised care and avoid diagnostic backlog to mitigate the impact of the COVID-19 pandemic.

From the public health perspective, our study had a unique opportunity to evaluate the impact of lockdown measures on stroke care independently of the COVID-19 surge. Due to early and efficient lockdown in Lithuania, the incidence and mortality of COVID-19 during the study period remained one of the lowest in Europe [22]. Second, to limit the potential spread of SARS-CoV-2 infection, the Lithuanian government implemented a strict state-wide lockdown, severely restricting social life and access to routine healthcare services [15]. Therefore, we could successfully examine the impact of lockdown measures on stroke care not confounded by the COVID-19 surge effect on the healthcare system. Our findings suggest that the state-wide lockdown measures could have had unwanted collateral consequences on prehospital stroke care.

Our study’s strengths include a prospective design and being the first report on the impact of state-wide lockdown on prehospital stroke triage quality and stroke mimics distribution. In addition, a large representative population of an urban academic centre covering one-third of the entire country allows for the generalisability of our findings to similar healthcare systems.

Our study includes some limitations. Foremost, since this was a single-centre study, we cannot account for referral pattern changes in other stroke centres in the country. However, the confounding should not have been significant as all centres functioned in the same low-incidence, low-mortality COVID-19 setting. Another limitation was the absence of data on medium and long-term functional outcomes. Even though the discharge NIHSS scores did not differ before and during the lockdown, we could not evaluate if longer delays to ED presentation might have resulted in poorer long term functional outcome. In addition, we did not fully account for the natural seasonal changes in the incidence of stroke. However, in 2018–2019 more strokes were admitted to our CSC during the spring rather than winter months, suggesting an actual decrease of stroke admission in our study rather than a seasonal fluctuation. Finally, our findings have limited generalisability to high-incidence COVID-19 situations, regions with different stroke care protocols, and varying social and healthcare responses to the COVID-19 pandemic.

## 5. Conclusions

In this prospective analysis of prehospital stroke care in a large academic stroke centre, we found a decreased incidence of stroke alerts and stroke/TIA admissions, as well as a drop in prehospital stroke triage quality during the state-wide COVID-19 lockdown. Furthermore, we observed an increase in hospital arrival delays and the proportion of severe conditions presenting as stroke mimics. Our findings suggest that public concern for SARS-CoV-2 infection and the state-wide lockdown negatively affected prehospital stroke care independently of the COVID-19 surge. These data are essential for policymakers to drive change in stay-at-home messaging, emphasising the importance of rapid transport to the hospital for acute conditions and improving access to neurological care during public health emergencies. Future studies should evaluate the impact of COVID-19 lockdown on functional stroke outcomes and stroke referral patterns at a national level.

## Figures and Tables

**Table 1:** Demographic and clinical characteristics of all stroke alerts.

	All Patients (*n* = 719)		Referred by EMS (*n* = 493)		Referred by A Physician (*n* = 226)		*p*-Value	Before Lockdown (*n* = 468)		During Lockdown (*n* = 251)		*p*-Value
Female, *n* (%)	431	59.9	286	58	145	64.2	0.118	280	59.8	151	60.2	0.931
Mean age, years (SD)	72.3	13.3	73	12.4	70.7	14.9	0.139	72.2	13.5	72.4	12.9	0.894
All strokes, *n* (%)	472	65.6	378	76.7	94	41.6		304	65.0	168	66.9	
Ischaemic stroke	397	55.2	321	65.1	76	33.6	<0.001	246	52.6	151	60.2	0.051
Haemorrhagic stroke	42	5.8	36	7.3	6	2.7	0.014	31	6.6	11	4.4	0.222
ICH	36	5	31	6.3	5	2.2	0.026	26	5.6	10	4.0	0.357
SAH	6	0.8	5	1.0	1	0.4	0.671	5	1.1	1	0.4	0.671
Transient ischaemic attack	33	4.6	21	4.3	12	5.3	0.532	27	5.8	6	2.4	0.039
Stroke mimics, *n* (%)	247	34.4	115	23.3	132	58.4	<0.001	164	35	83	33.1	0.595
Daily volume, median (IQR)												
Stroke alerts	3	(2–5)	2	(1–4)	1	(0–2)	<0.001	4	(3–6)	3	(1–4)	<0.001
Confirmed strokes	2	(1–3)	2	(1–2)	0	(0–1)	<0.001	3	(2–4)	2	(1–2)	<0.001
Reperfusion, *n* (%) †	108	27.2	101	31.5	7	9.2		66	26.8	42	27.8	
Not eligible	289	72.8	220	68.5	69	90.8	<0.001	180	73.2	109	72.2	0.830
IVT	50	12.6	49	15.3	1	1.3	<0.001	33	13.4	17	11.3	0.530
EVT	40	10.1	35	10.9	5	6.6	0.395	24	9.8	16	10.6	0.787
Combined treatment	18	4.5	17	5.3	1	1.3	0.217	9	3.7	9	6.0	0.285
Median timeliness metrics, min (IQR)												
Onset−to−door ‡	138.5	(82–274)	116	(76–245)	245	(145–378)	<0.001	120.5	(79–247)	153	(89–329)	0.049
Door−to−needle	37	(25–51)	37	(26–50)	35.5	(-)	0.790	39.5	(25–51)	35	(27–46)	0.557
Door−to−groin	86	(65–103)	86	(66–103)	83.5	(60–112)	0.918	85	(60–107)	87	(68–103)	0.946
Baseline NIHSS, median (IQR) §	7	(4–15)	8	(4–15)	6.5	(3–12)	0.175	8	(4–16)	7	(4–14)	0.415
Missing NIHSS, *n* (%)	167	42.1	113	35.2	54	71.1		110	44.7	57	37.7	
Discharge NIHSS, median (IQR) §	3	(1–6)	3	(1–6)	2.5	(0–5)	0.501	3	(1–6)	3	(1–5)	0.914
Missing NIHSS, *n* (%)	267	67.3	203	63.2	64	84.2		168	68.3	99	65.6	

EMS, emergency medical services; SD, standard deviation; ICH, intracerebral haemorrhage; SAH, subarachnoid haemorrhage; IQR, interquartile range; IVT, intravenous thrombolysis; EVT, endovascular treatment; NIHSS, National Institutes of Health Stroke Scale. † Percentage of all ischaemic strokes. ‡ Only patients with established onset of symptoms are included (*n* = 282). § NIHSS is reported only for ischaemic stroke patients who were considered for reperfusion therapy.

**Table 2:** Positive predictive values (PPV) for the identification of patients with acute stroke by the Emergency Medical Services (EMS) and referring physicians.

	Total PPV (95% CI)	PPV before Lockdown (95% CI)	PPV during Lockdown (95% CI)	*p*-Value
Referred by				
EMS	76.7% (72.9–80.4) (*n* = 493)	79.9% (75.3–84.5) (*n* = 289)	72.1% (65.9–78.2) (*n* = 204)	0.042
Physician	41.6% (35.2–48.0) (*n* = 226)	40.8% (33.6–48.0) (*n* = 179)	44.7% (30.5–58.9) (*n* = 47)	0.629
*p*-value	<0.001	<0.001	<0.001	

CI, confidence interval.

**Table 3:** Most common stroke mimics.

Stroke Mimic, *n* (%)	All Mimics (*n* = 247)		Referred by EMS (*n* = 115)		Referred by A Physician (*n* = 132)		*p*-Value	before Lockdown (*n* = 164)		during Lockdown (*n* = 83)		*p*-Value
Seizure	25	(10.1)	19	(16.5)	6	(4.5)	0.002	11	(6.7)	14	(16.9)	0.012
Peripheral vestibulopathy	25	(10.1)	1	(0.9)	24	(18.0)	<0.001	21	(12.8)	4	(4.8)	0.072
Hypertensive encephalopathy	23	(9.3)	11	(9.6)	12	(9.1)	0.898	17	(10.4)	6	(7.2)	0.423
Infection/Sepsis	22	(8.9)	16	(13.9)	6	(4.5)	0.010	13	(7.9)	9	(10.8)	0.447
Toxic/Metabolic disorder †	18	(7.3)	9	(7.8)	9	(6.8)	0.761	9	(5.5)	9	(10.8)	0.126
Sequels of previous stroke	16	(6.5)	5	(4.3)	11	(8.3)	0.300	11	(6.7)	5	(6.0)	1.000
Intracranial tumour	13	(5.3)	9	(7.8)	4	(3.0)	0.151	5	(3.0)	8	(9.6)	0.037
Psychiatric condition ‡	13	(5.3)	7	(6.1)	6	(4.5)	0.588	11	(6.7)	2	(2.4)	0.153
Migraine and headache	10	(4.0)	4	(3.5)	6	(4.5)	0.755	8	(4.9)	2	(2.4)	0.502
Cardiovascular condition §	10	(4.0)	4	(3.5)	6	(4.5)	0.755	6	(3.7)	4	(4.8)	0.736
Syncope and presyncope	8	(3.2)	2	(1.7)	6	(4.5)	0.291	7	(4.3)	1	(1.2)	0.273
Facial nerve palsy	5	(2.0)	3	(2.6)	2	(1.5)	0.666	2	(1.2)	3	(3.6)	0.338
Musculoskeletal condition	5	(2.0)	2	(1.7)	3	(2.3)	1.000	3	(1.8)	2	(2.4)	1.000
Subdural haematoma	4	(1.6)	3	(2.6)	1	(0.8)	0.341	4	(2.4)	0		0.304
Peripheral neuropathy	4	(1.6)	1	(0.9)	3	(2.3)	0.626	3	(1.8)	1	(1.2)	1.000
Isolated symptoms associated with normal results ¶	21	(8.5)	6	(5.2)	15	(11.4)	0.084	19	(11.6)	2	(2.4)	0.015
Other neurological disease ||	20	(8.1)	10	(8.7)	10	(7.6)	0.748	12	(7.3)	8	(9.6)	0.528
Other non−neurological disease #	5	(2.0)	3	(2.6)	2	(1.5)	0.666	2	(1.2)	3	(3.6)	0.338

EMS, Emergency Medical Services; CNS, central nervous system. † Includes electrolyte imbalances (*n* = 14), hypo- and hyperglycaemia (*n* = 2), hepatic encephalopathy (*n* = 1), and alcohol intoxication (*n* = 1). ‡ Panic attacks and anxiety (*n* = 8), as well as delirium (*n* = 5)). § Cardiac insufficiency (*n* = 4), cardiac arrhythmia (*n* = 2), acute myocardial infarction (*n* = 2), and pulmonary embolism (*n* = 2). ¶ Neurological symptoms that could not be classified into any other category since they did not meet any other set of diagnostic criteria or which have no organic explanation but are not clearly associated with a psychiatric disorder. || Includes multiple sclerosis and other demyelinating diseases (*n* = 5), dementia (*n* = 4), transient global amnesia (*n* = 4), myelopathy (*n* = 2), CNS infection (*n* = 2), venous sinus thrombosis (*n* = 1), Parkinson’s disease (*n* = 1), and myasthenia (*n* = 1). # Includes advanced cancer (*n* = 2), anemia (*n* = 2), and herpes zoster (*n* = 1).

## Data Availability

The data that support the findings of this study are available from the corresponding author upon reasonable request.
